# Supplementary material for: Imaging the columnar functional organization of human area MT+ to axis-of-motion stimuli using VASO at 7 Tesla
Source: Cereb Cortex. 2023 May 30;33(13):8693–711. doi: 10.1093/cercor/bhad151 (PMC10321107; doi:10.1093/cercor/bhad151)
Supplement: 2022_Preprint_AOM_V_04_SupplementalMatherial-36-39_bhad151 [file 2022_preprint_aom_v_04_supplementalmatherial-36-39_bhad151.pdf]

Supplementary Material

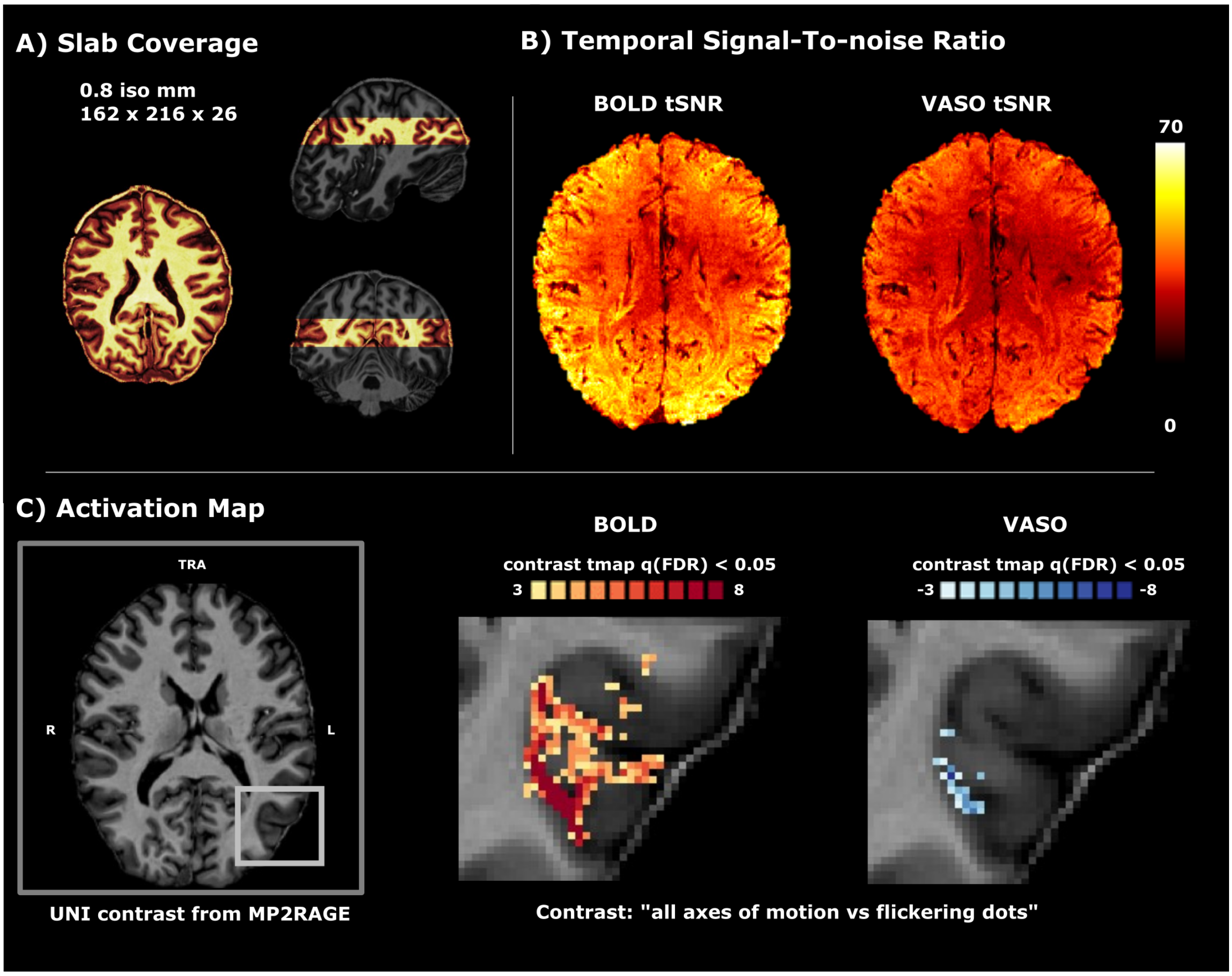

Supplementary Figure 1: SS-SI VASO functional images for an example participant (sub-01). A) Slab coverage (in warm colors) relative to a whole brain MP2RAGE. B) Temporal signal-to-noise ratio. C) Activation map for BOLD and VASO time series for the contrast: "all axes of motion vs flickering dots" (q(False Discovery Rate) < 0.05). for the left-hMT+.

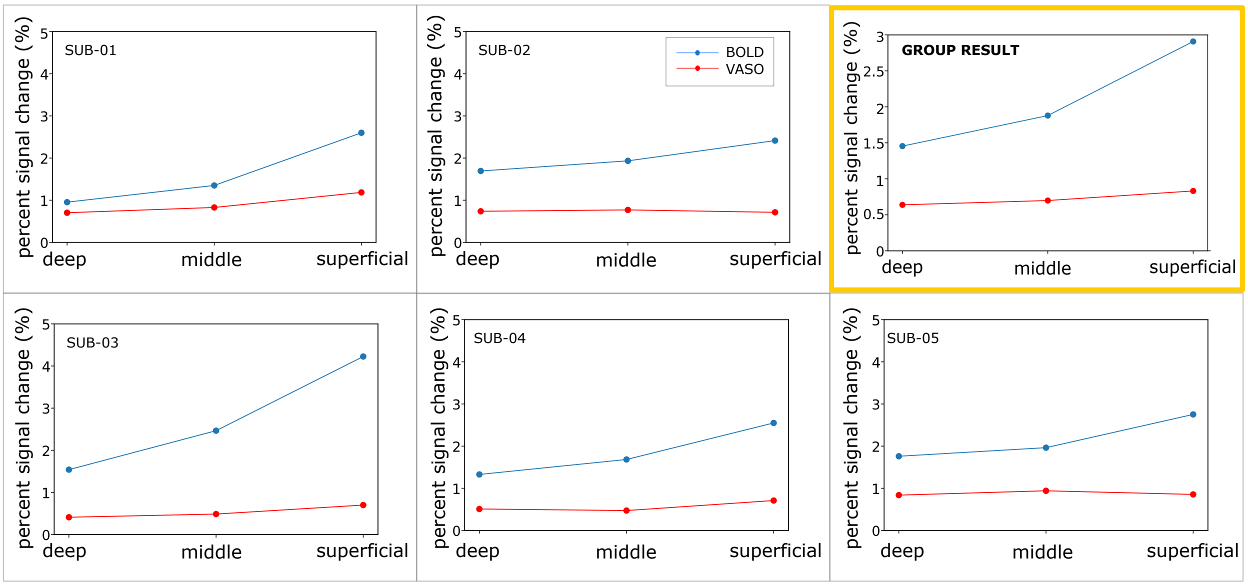

Supplementary Figure 2: Layer profiles across three cortical depths: deep (25%), middle (50%), superficial (75%) with respect to the white-gray matter boundary in the left hMT+. For each cortical depth, the averaged percent signal change of all cross-validated voxels is reported for both BOLD and VASO. The yellow box shows the group result of all five participants. See **Supplementary Figure 3A** for a detailed version of this figure showing individual participants and conditions.

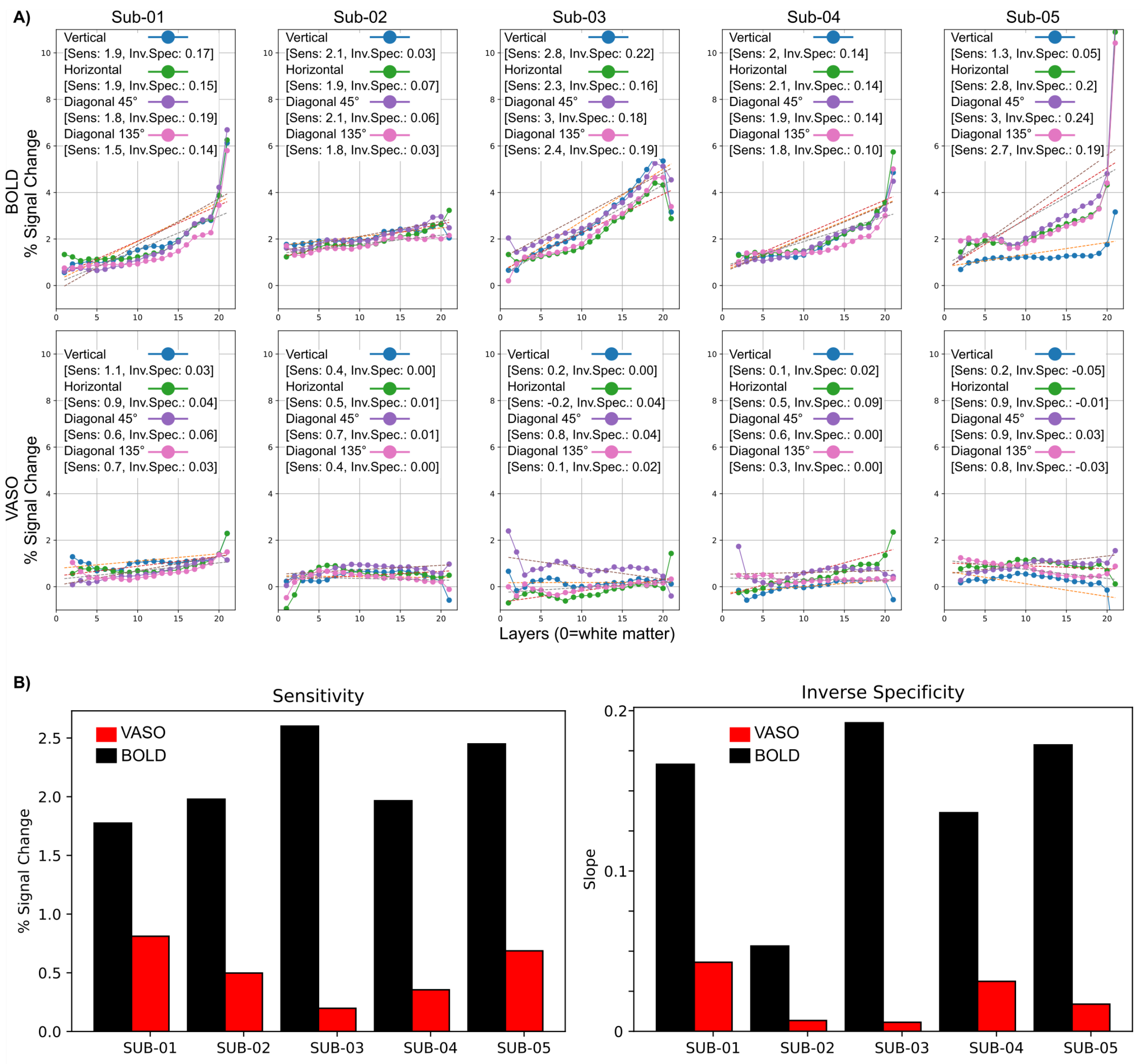

Supplementary Figure 3: Global metrics of sensitivity and inverse specificity computed for each subject (columns), for both BOLD (first row) and VASO contrast (second row) considering cross-validated voxels in the left hMT+. Each subplot shows single layer profiles for each axis-of-motion condition. The square brackets report global sensitivity and inverse measure of global specificity (Beckett et al., 2020). Condensed version of this panel showing average stimulation conditions can be seen in **Supplementary Figure 2**. B) Global metrics of sensitivity and inverse specificity (Beckett et al., 2020) computed for each participant, for both BOLD (black bar) and VASO (red bar). Sensitivity is calculated by averaging signal change across layers. The inverse specificity is measured by the slope of a line fit to each depth profile. Each barplot depicts the mean value evaluated across the four axes of motion.

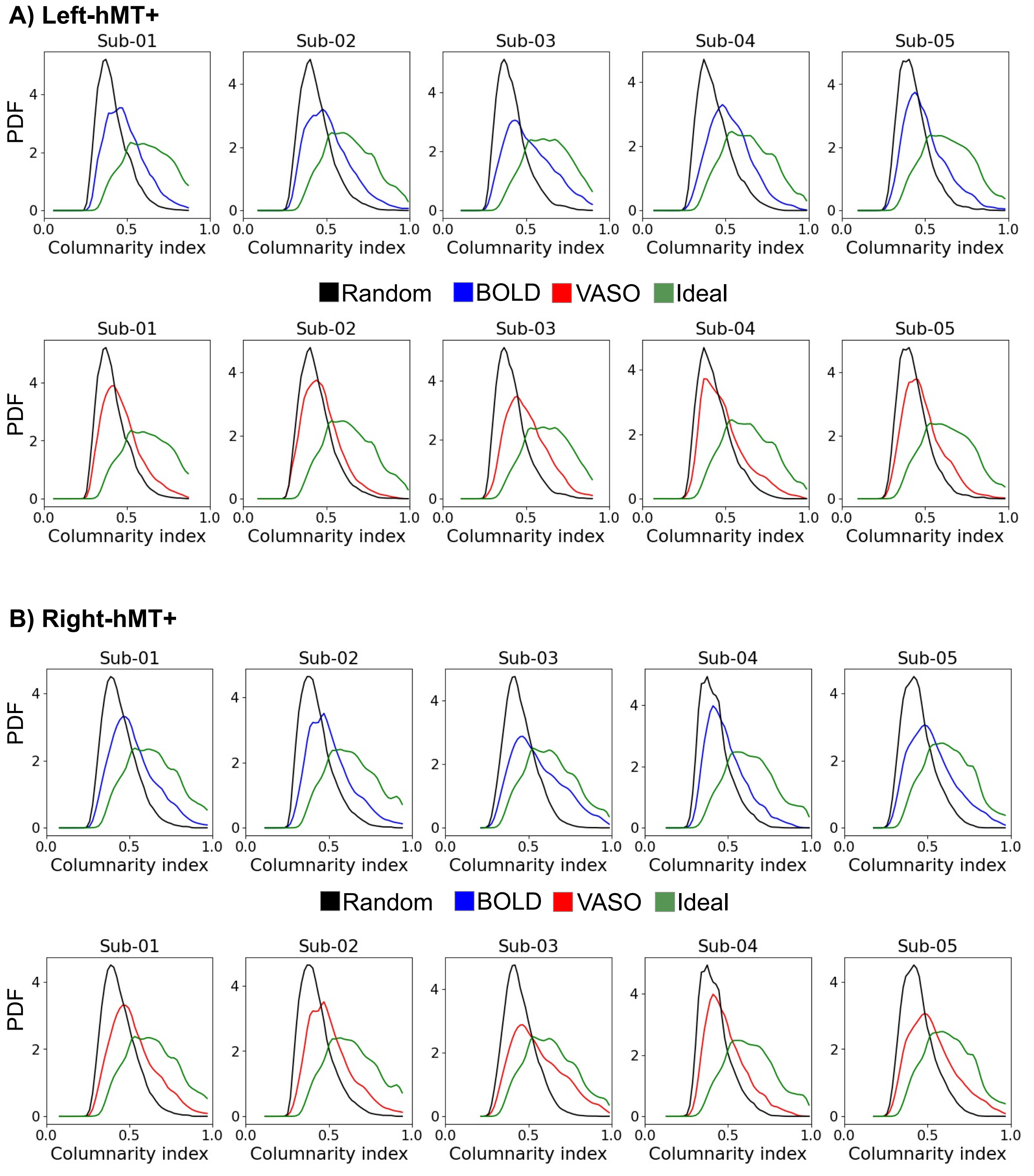

Supplementary Figure 4: Comparing columnarity's index probability density function (PDF) of empirical and benchmark data for each participant. First rows of panel A and B show BOLD (blue color), 'random' and 'ideal' PDFs. Second rows of panel A and B show VASO (red color), 'random' and 'ideal' PDFs. Condensed version of this figure showing group results can be seen in **Figure 10**.
